# Decoding the contributions of gut microbiota and cerebral metabolism in acute liver injury mice with and without cognitive dysfunction

**DOI:** 10.1111/cns.14069

**Published:** 2022-12-30

**Authors:** Tianning Sun, Hongying Du, Zhen Li, Jun Xiong, Yanbo Liu, Yujuan Li, Wencui Zhang, Fangyuan Liang, Jingang He, Xiaodong Liu, Hongbing Xiang

**Affiliations:** ^1^ Department of Anesthesiology, Tongji Hospital, Tongji Medical College Huazhong University of Science and Technology Wuhan China; ^2^ Department of Food Science and Engineering, College of Light Industry and Food Engineering Nanjing Forestry University Nanjing China; ^3^ Hepatobiliary Surgery Center, Union Hospital, Tongji Medical College Huazhong University of Science and Technology Wuhan China; ^4^ College of Acupuncture and Orthopedics Hubei University of Chinese Medicine Wuhan China; ^5^ Key Laboratory of Magnetic Resonance in Biological Systems, State Key Laboratory of Magnetic Resonance and Atomic and Molecular Physics, National Center for Magnetic Resonance in Wuhan, Wuhan Institute of Physics and Mathematics, Innovation Academy for Precision Measurement Science and Technology Chinese Academy of Sciences‐Wuhan National Laboratory for Optoelectronics Wuhan China; ^6^ Department of Anaesthesia and Intensive Care, Peter Hung Pain Research Institute The Chinese University of Hong Kong Hong Kong China

**Keywords:** acute liver injury, cerebral neurotransmitter metabolic, cognitive dysfunction, microbiota, synaptic transmission

## Abstract

**Aims:**

Patients with acute liver injury (ALI) can develop cognitive dysfunction (CD). The study investigated the role of gut microbiota and cerebral metabolism in ALI mice with and without CD.

**Methods:**

Male C57BL/6 mice that received thioacetamide were classified into ALI mice with (susceptible) or without (unsusceptible) CD‐like phenotypes by hierarchical cluster analysis of behavior. The role of gut microbiota was investigated by 16S ribosomal RNA gene sequencing and feces microbiota transplantation (FMT). ^1^H‐[^13^C] NMR and electrophysiology were used to detect the changes in cerebral neurotransmitter metabolic and synaptic transition in neurons or astrocytes.

**Results:**

Apromixlay 55% (11/20) of mice developed CD and FMT from the susceptible group transmitted CD to gut microbiota‐depleted mice. *Alloprevotella* was enriched in the susceptible group. GABA production was decreased in the frontal cortex, while hippocampal glutamine was increased in the susceptible group. Altered *Escherichia. Shigella* and *Alloprevotella* were correlated with behaviors and cerebral metabolic kinetics and identified as good predictors of ALI‐induced CD. The frequencies of both miniature inhibitory and excitatory postsynaptic currents in hippocampal CA1 and prefrontal cortex were decreased in the susceptible group.

**Conclusion:**

Altered transmitter metabolism and synaptic transmission in the hippocampus and prefrontal cortex and gut microbiota disturbance may lead to ALI‐induced CD.

## Graphical abstract

The gut microbiota composition and cerebral transmitter metabolism differ in acute liver injury mice with and without cognitive impairment. The *Alloprevotella* and *Escherichia.Shigella* related to transmitter metabolism increase in the cognitive dysfunction mice. The GABA_2_ enrichment decreases in the frontal cortex and the glutamine_4_ enrichment increases in the hippocampus, which induces dysregulated synaptic transmission in the cognitive dysfunction mice.

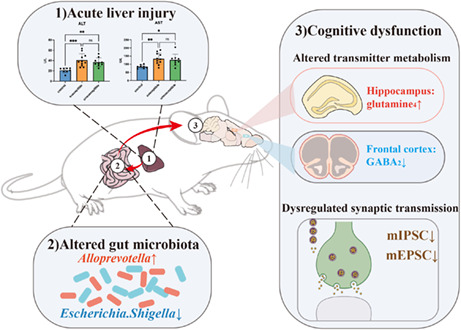

## 1 INTRODUCTION

Acute liver injury (ALI) refers to the impaired liver function that occurs in a short period of time and is mainly manifested by liver cell necrosis and elevation of hepatic enzymes in the blood.^1^ The ALI can be caused by a variety of insults, such as acetaminophen‐induced hepatotoxicity, drug‐induced liver injury, and hepatitis viral infection.^2^, ^3^ In the early stage of ALI, some patients may recover on their own, while others will even develop hepatic encephalopathy (HE) to varying degrees.^4^, ^5^ HE is associated with higher mortality and serves as a prognostic indicator for ALI.^6^, ^7^ Minimizing the progression of HE in ALI is critical to improving patient outcomes.^8^

The pathogenesis of HE is closely related to abnormal metabolism in the brain. Hyperammonemia and brain energy metabolism disorder may be responsible for the HE.^6^, ^9^ Studies are showing that increased levels of lactate and glutamine in the brain could lead to swelling of astrocytes in cirrhotic rats with HE.^10^, ^11^ Glutamine accumulation, dysregulated cerebral glucose metabolism, and changes in cerebral metabolism were also observed in the frontal lobe of comatose rats with acute liver failure.^12^, ^13^ Besides, altered glutamate and GABAergic neurotransmission is also reported to cause cognitive impairment in liver disease.^14^

Recently, microbiota–host interactions have been demonstrated in several liver diseases, including ALI and HE.^15^, ^16^, ^17^ Treatment and prevention strategies of ALI based on microbial modulation have been proposed.^18^ Microbiome‐targeted therapy, such as rifaximin^19^ and fecal microbiota transplantation (FMT), show encouraging therapeutic outcomes in patients with HE,^20^ although the mechanisms of action remain elusive. Thus, in this study, we investigated whether the shaping of the gut microbiota could affect cerebral metabolism and synaptic transmission and thereby regulate ALI‐associated cognitive dysfunction (CD).

## 2 MATERIALS AND METHODS

### 2.1 TAA‐induced acute liver injury

Male C57BL/6J mice (8 weeks) were purchased from Beijing Vital River Laboratory Animal Technology. All experiments were performed in accordance with national and EU guidelines and approved by the Institutional Animal Care and Use Committee of Tongji Hospital, Tongji Medical College, Huazhong University of Science and Technology (IRB ID: TJ‐A0803). Thioacetamide (TAA) purchased from Sigma‐Aldrich (PubChem ID: 24849761) was dissolved in saline at a concentration of 10 mg/ml. Mice in the ALI group were injected intraperitoneally (i.p.) with TAA at a dose of 50 mg/kg bodyweight for 4 consecutive days, and mice in the control group were injected with saline in the same volume.

### 2.2 Behavioral test

The open field test (OFT), Y‐maze test (Y‐maze), and novel object recognition test (NORT) were orderly performed to evaluate the cognition of mice. Animals' behavior was recorded by video cameras fixed on the ceiling and analyzed by ANY‐MAZE software. OFT: OFT device is made of a gray polyethylene box (L × W × H: 40 × 40 × 40 cm). Mice were placed in the center and allowed to move freely for 5 min. The total distance traveled was analyzed. NORT: Two identical objects were placed in an open field, 6 cm away from the walls,^21^ each mouse was allowed to explore freely for 5 min and then returned to the cage for 2 h. After one object was replaced with a novel object, mice were put back into the open field and allowed to explore freely for 5 min. The time mice spent exploring the novel object (TN) and the familiar object (TF) were recorded. The recognition index = TN/(TN + TF). Y‐maze: the Y‐maze device consisted of three arms at an angle of 120° each (L × W × H: 30 cm × 8 cm × 15 cm). In the first learning stage, mice were placed in the starting arm (randomly assigned), while the new arm (randomly assigned) was blocked and allowed to explore freely for 10 min. The mouse was then returned to the cage for 2 h. In the testing stage, mice were placed in the same starting arm (the new arm was open) and allowed to explore for 5 min. The time spent on each arm was analyzed

### 2.3 Fecal samples collection and Fecal microbiota transplantation (FMT)

Fecal samples were collected after all behavioral tests. Immediately after defecation, fecal samples were collected and stored at −80°C. FMT was applied as described before.^21^ Briefly, mice were treated with water containing broad‐spectrum antibiotics (1 g/L: Metronidazole; Ampicillin; Neomycin Sulfate; and Vancomycin 0.5 mg/kg) for 2 weeks to eliminate gut microbes. Then, the antibiotics were removed 24 h before FMT. Feces collected from the three groups were resuspended in PBS (phosphate‐buffered solution) at 0.2 g/ml. Gut microbiota‐depleted mice were infused intragastrically with 0.2 ml of suspension or PBS once daily for 14 days.

### 2.4 Tissue harvest and hematoxylin and eosin (H&E) staining

Mice were deeply anesthetized with sodium pentobarbital (*i*.*p*. 100 mg/kg) and rapidly decapitated to harvest blood. The upper serum was sent to the clinical laboratory of Tongji Hospital for the determination of total bilirubin (TBIL), alanine aminotransferase (ALT), and aspartate aminotransferase (AST) levels. The left lobe of the liver was fixed in a 4% neutral formaldehyde solution. The specimen was embedded in paraffin and sliced into 5 μm thick sections, then stained with hematoxylin and eosin. The liver damage was estimated by histological activity index analysis.^22^

### 2.5 16S rRNA gene sequencing

16S rRNA gene sequencing of fecal samples was performed at OEBiotech Co. Ltd. DNA was extracted using DNA Extraction Kit (Tiangen Biotechnology Co., Ltd.). V3‐V4 variable regions of 16S rRNA genes were amplified with universal primers 343 F and 798 R. Purified PCR products were used for sequencing. All representative reads were annotated and blasted against Unite database (ITSs rDNA).

### 2.6 Sample preparation for^1^H‐[^13^C] NMR study

According to our previous research.^23^, ^24^ Fasted overnight (15–18 h), mice were infused with [1‐^13^C] glucose (Qingdao Tenglong Weibo Technology Co., Ltd.) via the tail vein. After 30 min, mice were deeply anesthetized with isoflurane and rapidly decapitated, and the animal head was microwaved (0.75 kw, 15 s) to stop the metabolism of the brain. The brain was dissected into nine regions, namely frontal cortex (FC), parietal cortex (PC), occipital cortex (OC), temporal cortex (TC), striatum (STR), hippocampus (HIP), thalamus (TH), medulla‐pons (PON), and cerebellum (CE).^25^

Brain metabolites extraction was done according to our previous studies.^25^ Firstly, HCL/methanol (200 μl, 0.1 M) solution was added to the brain tissues. Secondly, 800 μl ethanol (60%, vol/vol) was added, and the samples were homogenized, centrifuged at 18966 *g* for 10 min, and the supernatant was collected. Repeat the second step three times. After evaporated and lyophilized, the lyophilizate was dissolved in a D_2_O buffer solution (600 μl of D_2_O containing the internal standard, 3‐(trimethylsilyl) propionic‐2, 2, 3, 3‐d_4_ acid sodium salt (TMSP, 5 mM; 269913‐1G, Sigma‐Aldrich) and 0.2 M Na_2_HPO_4_/NaH_2_PO_4_, pH = 7.2). After centrifugation at 18966 g for 15 min, 550 μl of the supernatant was transferred to a 5 mm NMR tube for [^1^H‐^13^C]‐NMR analysis.

### 2.7 NMR spectrum acquisition and data processing

[^1^H‐^13^C]‐NMR spectra were obtained at 298 K with a BrukerAvance III 500 MHz NMR spectrometer (BrukerBioSpin, Germany) (acquisition parameters: a number of scans‐64; repetition time‐20 sec; sweep width‐20 ppm; acquisition data‐64 K; echo time‐8 ms). Phase correction and baseline distortion were manually completed in Topspin 2.1 (Bruker Biospin, GmbH). The^13^C enrichment of metabolites was automatically calculated with a homemade software NMRSpec^26^ in MATLAB (Freely available from the author upon request: jie.wang@wipm.ac.cn).

### 2.8 Whole‐cell patch‐clamp recording

Mice were anesthetized with sodium pentobarbital (*i*.*p*. 100 mg/kg) and rapidly decapitated. PrL (prelimbic cortex) and hippocampus CA1 slices (350 μm) were obtained by a vibrating microtome (VT1000S, Leica, Germany) in dissection solution at 4°C (mM: 213 sucrose, 3 KCl, 1 NaH_2_PO_4_, 0.5 CaCl_2_, 5 MgCl_2_, 26 NaHCO_3_, and 10 glucose). After 1 h incubation at 34°C in artificial cerebrospinal fluid (ACSF) (mM: 125 NaCl, 5 KCl, 1.2 NaH_2_PO_4_, 2.6 CaCl_2_, 1.3 MgCl_2_, 26 NaHCO_3_, and 10 glucose), a single brain slice was transferred onto the recording chamber and perfused with running 37°C ACSF equilibrated with 95% O_2_ and 5% CO_2_ at a rate of 2 ml/min. Only 1–2 brain slices per animal were used, and only 1 neuron was recorded in each slice.

Miniature inhibitory postsynaptic currents (mIPSCs) and miniature excitatory postsynaptic currents (mEPSCs) was recorded according to our previous research.^27^ Patch pipettes filled with the intracellular solution (145 KCl, 5 NaCl, 10 HEPES, 5 EGTA, 4 Mg‐ATP, and 0.3 Na3‐GTP (in mM)). Data were acquired and analyzed using the following equipment: HEKA EPC‐10 amplifier (HEKA), PATCHMASTER software (Molecular Devices), a dual four‐pole Bessel filter (Warner Instruments), a low‐noise Digidata 1322 interface (Molecular Devices), and a Pentium PC. The mIPSCs were recorded in the presence of dl‐2‐amino‐5‐phosphonopentanoic acid (AP‐5) (50 μm) and 2,3‐dioxo‐6‐nitro‐1,2,3,4‐tetrahydrobenzo[f]quinoxaline‐7‐sulfonamide disodium salt (NBQX) (20 μm) at a holding potential of −40 mV. The mEPSCs were recorded in the presence of 50 μM picrotoxin and 1 μM tetrodotoxin at a holding potential of −70 mV. The mIPSCs and mEPSCs were measured 10 min before and 15 min after drug application. A fixed length of traces (5 min) was analyzed for frequency and amplitude distributions with Mini Analysis Program 6.0 (Synaptosoft Inc) and pCLAMP10 software.

### 2.9 Statistical analysis

Statistical analyses were performed using SPSS 17.0 (IBM) and GraphPad Prism 8.0. Y‐maze results were hierarchically clustered by using the Ward method and Euclidean distance square as distance measurement and standardizing with the Z‐score.^21^ Data were analyzed by one‐way analysis of variance, followed by post hoc Bonferroni's test. *p* < 0.05 was considered to indicate a statistically significant difference. The correlation analysis was performed using the psych package in the R environment and displayed by Cytoscape 3.9.1. The module of the correlation network was identified by Molecular Complex Detection (MCODE) plugins. Hub candidates were identified by CytoHubba using Maximal Clique Centrality (MCC).

## 3 RESULTS

### 3.1 Effect of acute liver injury on cognitive function

**FIGURE 1:**
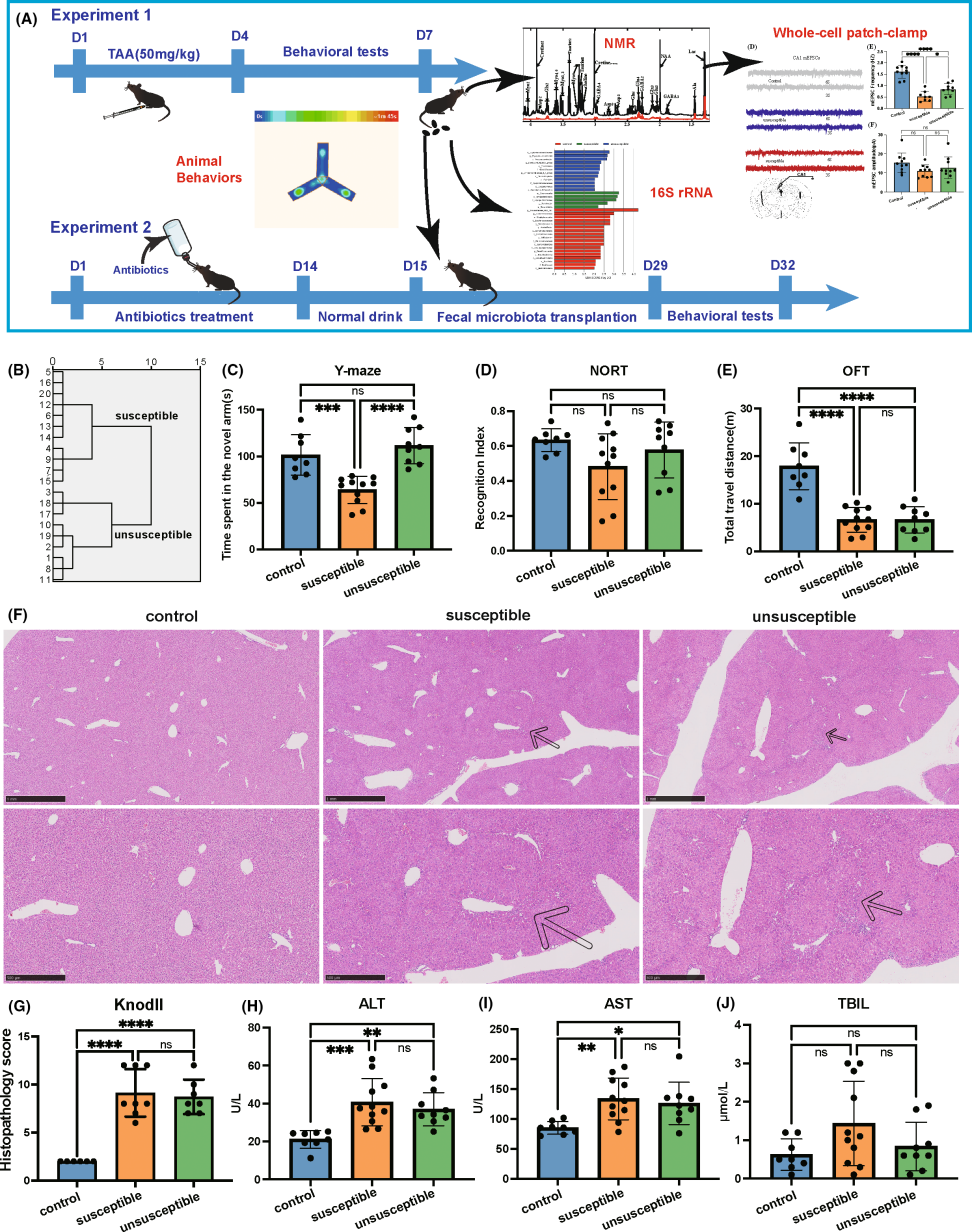
ALI induced by TAA causes cognitive dysfunction in mice. (A) On Experiment 1, TAA was injected intraperitoneally into mice at 50 mg/kg for 4 days. Behavioral tests (OFT, NORT, and Y‐maze, respectively) were performed in the last 3 days. Mice that received TAA were divided into the susceptible and unsusceptible groups by hierarchical cluster analysis of the Y‐maze test. On Day 7, fecal samples were collected for FMT and 16S rRNA sequencing. Brain tissues were collected to perform NMR analysis and electrophysiology. Then, the correlation between microbiota, NMR, and behaviors was analyzed. On Experiment 2, mice were treated with antibiotics for 14 days. Fecal microbiotas from Experiment 1 were transplanted to mice. And behavioral tests were conducted on Days 29–32. (B) Dendrogram of hierarchical clustering analysis of the behavioral test. A total of 20 TAA mice are divided into susceptible (*n* = 11) and unsusceptible (*n* = 9) groups by hierarchical clustering analysis of Y‐maze. (C) Y‐maze test on Day 7 (*F*_2,25_ = 18.81, *p* < 0.0001). (D) Recognition Index of NORT (*F*_2,25_ = 2.402, *p* = 0.1111). (E) Total travel distance of OFT (*F*_2,25_ = 30.4, *p* < 0.0001). (F) H&E staining of the liver. (G) Pathological scores of liver injury (*F*_2,18_ = 29.85, *p* < 0.0001). (H) Serum ALT levels (*F*_2,25_ = 10.33, *p* = 0.0005). (I) Serum AST levels (*F*_2,25_ = 6.435, *p* = 0.0056). (J) Serum TBIL levels (*F*_2,25_ = 2.636, *p* = 0.0951). ALT, alanine aminotransferase; AST, aspartate aminotransferase; NORT, Novel Object Recognize Test; OFT, open field test;TAA, thioacetamide; TBIL, total bilirubin; Y‐maze, Y‐maze test. Data are presented as the mean ± SEM. *p < 0.05; **p < 0.01; ***p < 0.001; ****p < 0.0001; ns, not significant.

Schematic diagram of the experimental design is illustrated in Figure 1A. Seven days after the first TAA injection, the animals (*n* = 20) manifested differential spatial learning and memory as evidenced by the different time spent in the novel arm in the Y‐maze test, indicating that some mice had developed CD after ALI. We then divided animals into the mice with CD (susceptible, *n* = 11) and mice without CD (unsusceptible, *n* = 9) by the hierarchical cluster analysis of the Y‐maze test results (Figure 1B). The susceptible group, but not the unsusceptible group spent significantly less time in the novel arm than the control group (Figure 1C). But ALI did not affect short‐term memory (Figure 1D). The total travel distance of the susceptible and unsusceptible groups showed a downward trend, indicating that the mice's spontaneous movement decreased in these two groups (Figure 1E). Moreover, there were no differences in the total arm entries among the control, susceptible, and unsusceptible groups (Figure S1A). The susceptible only manifested decreased novel arm entries while identical start arm, and familiar arm entries compared with the control and unsusceptible groups (Figure S1B). These results suggested that the overall exploration of the Y‐maze of the three groups is identical and is not affected by reduced locomotor activity. Spatial memory impairment is raised by the reduced exploration of the novel arm of the susceptible group. Both susceptible and unsusceptible groups exhibited inflammatory cell immersion, bridge necrosis, and punctate necrosis in the livers (Figure 1F,G). In addition, serum ALT and AST levels of susceptible and unsusceptible groups are significantly higher than in the control group (Figure 1H,I).

### 3.2 ALI‐induced cognitive dysfunction is transmissible to intact mice by FMT

**FIGURE 2:**
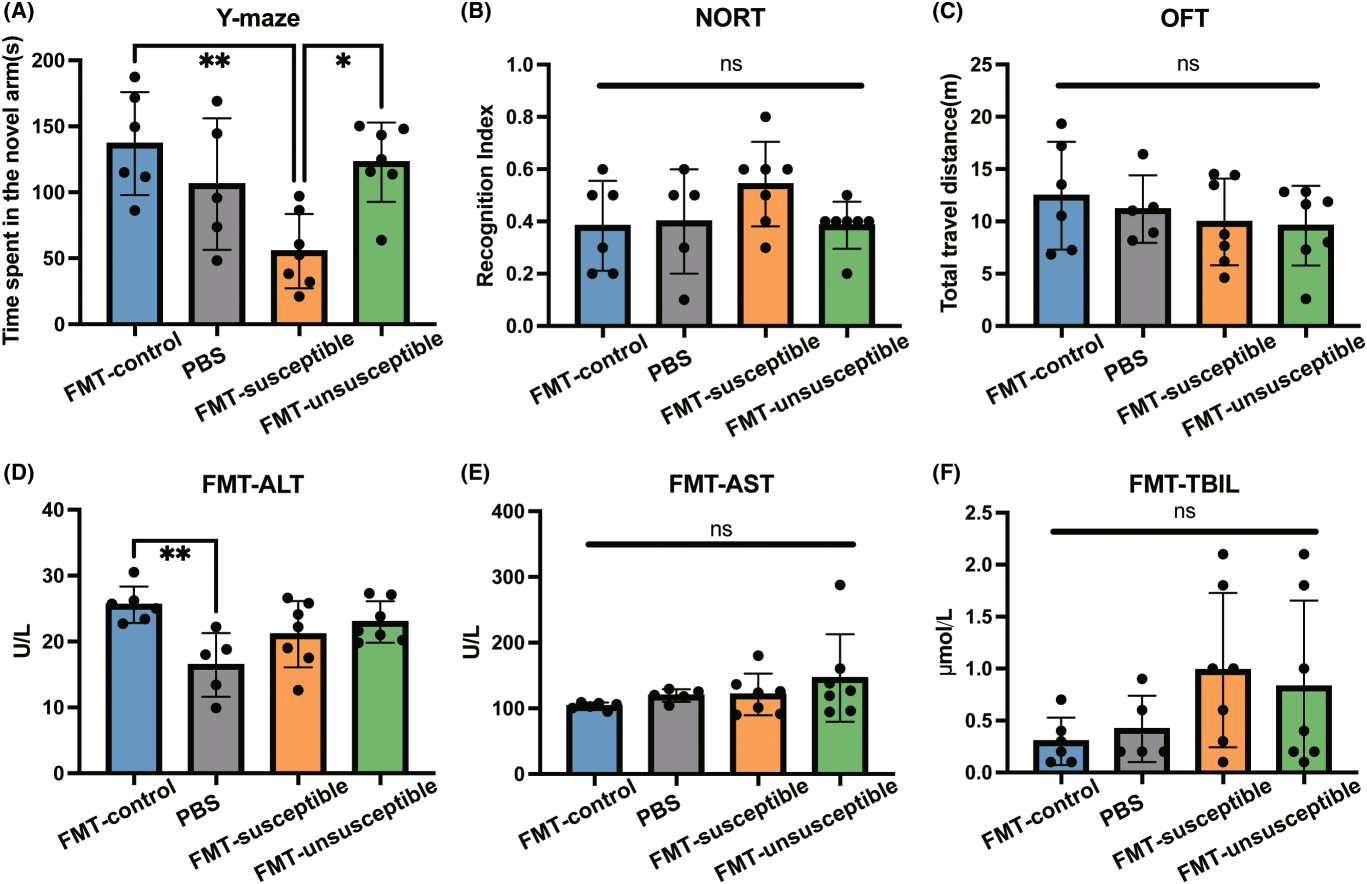
Effect of FMT from ALI mice on cognitive function in gut microbiota‐depleted mice. (A) Total travel distance of FMT mice (*F*_3,21_ = 0.6201, *p* = 0.6098). (B) Recognition Index of FMT mice (*F*_3,21_ = 1.639, *p* = 0.2106). (C) Y‐maze test of FMT mice (*F*_3,23_ = 6.449, *p* = 0.0029). (D) Serum ALT levels of FMT mice (*F*_3,23_ = 4.985, *p* = 0.0083). (E) Serum AST levels of FMT mice (*F*_3,23_ = 1.336, *p* = 0.2870). (F) Serum TBIL levels of FMT mice (*F*_3,23_ = 0.6041, *p* = 0.6190). NORT, Novel Object Recognize Test; OFT, open field test; TAA, thioacetamide; Y‐maze, Y‐maze test. Data are presented as the mean ± SEM. *p < 0.05; **p < 0.01; ns, not significant.

In order to assess the role of fecal microbiota for cognition, gut microbiota‐depleted mice were transplanted with stool suspensions from different groups of mice. PBS gavage was applied in the FMT‐free group. Compared to FMT‐control mice (transplanted with control stool) and FMT‐unsusceptible mice (transplanted with unsusceptible group stool), FMT‐susceptible mice (transplanted with susceptible group stool) spent significantly less time in the novel arm (Figure 2A). There was no significant difference in the NORT and OFT tests between these four groups (Figure 2B,C). Besides, there was no significant difference in the serum ALT, AST, and TBIL levels, which indicated that changes in cognitive behaviors were not caused by liver injury after FMT (Figure 2D–F). These results showed that the gut microbiota encompasses risk factors that could lead to ALI‐associated CD phenotype. Thus, we performed a 16S rRNA sequencing to examine the difference in fecal microbiota among control, susceptible, and unsusceptible groups.

### 3.3 Alternation of fecal microbiota in ALI mice with or without cognitive dysfunction

**FIGURE 3:**
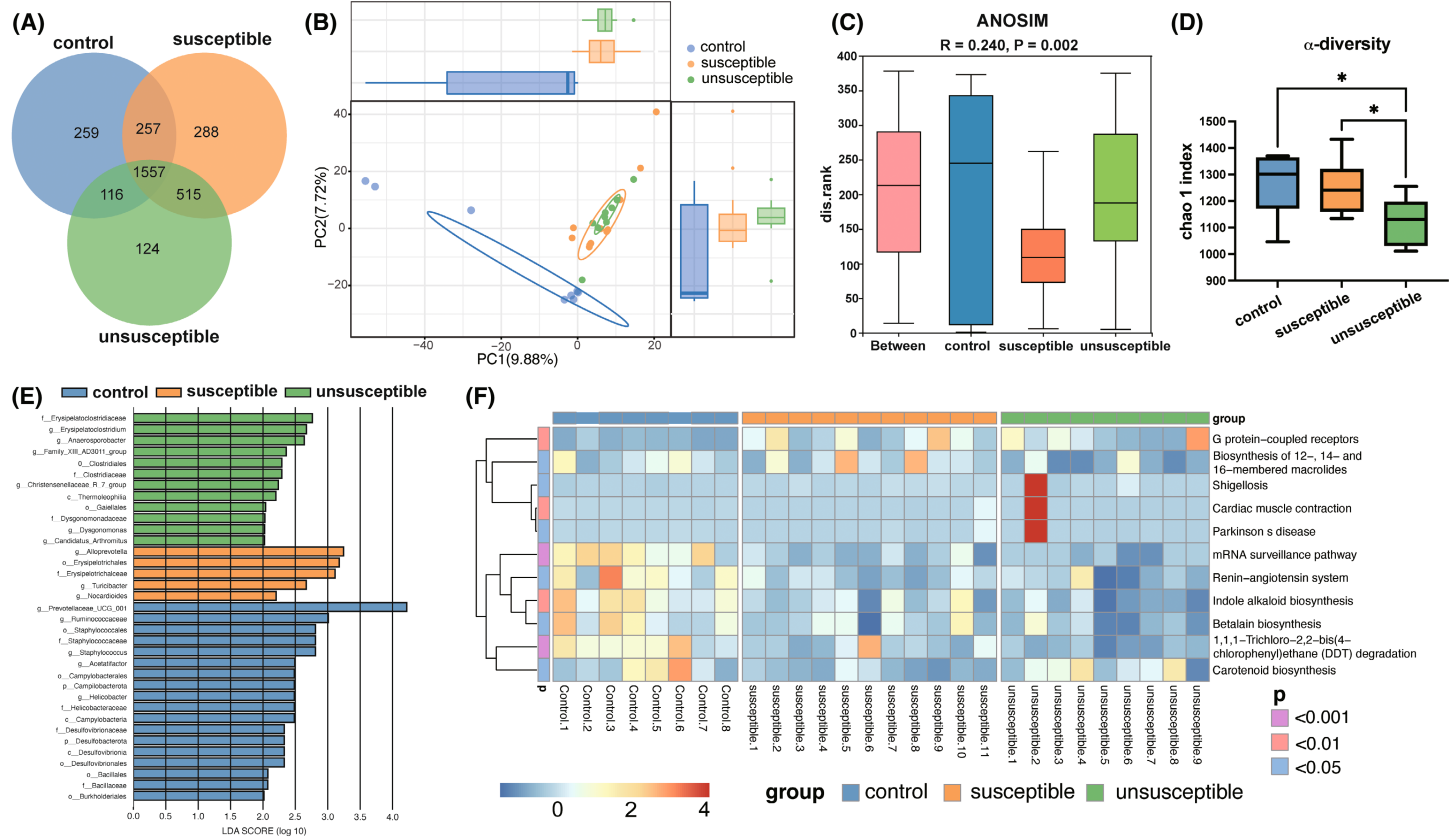
16S rRNA analysis of the fecal microbiota in ALI with or without cognitive dysfunction. (A) Venn diagram of groups. (B) PCA analysis of β‐diversity. (C) Bray–Curtis ANOSIM analysis. (D) α‐diversity chao1 index (*F*_2,25_ = 4.782 *p* = 0.0174). (E) Difference species score chart obtained using linear discriminant analysis (LDA) effect size (LEfSe) analysis. The blue, orange, and green stripes represent the species with relatively high abundance in the control group, susceptible group, and unsusceptible group individually. (F) KEGG pathway prediction heat map based on 16S. Note: The letters before the microbiota name represent the different species levels, p: phylum; c: class; o: order; f: family; g: genus. Data are shown as mean ± S.E.M; *p < 0.05, ns, not significant.

The Venn diagram shows that 259, 188, and 125 OTUs are unique to control, susceptible, and unsusceptible groups, respectively, revealing a great difference among the three groups (Figure 3A). Principal component analysis (PCA) demonstrated a clean separation between the control and ALI mice (Figure 3B,C, ANOSIM: *R* = 0.385, *p* = 0.002). For two ALI groups, the unsusceptible group has a significantly lower Chao 1 index (alpha‐diversity) than the susceptible group (Figure 3D). LEfSe analysis shows that the increased abundance of *g__Erysipelatoclostridium*, and *f__Erysipelatoclostridiaceae* in the unsusceptible group; *f__Erysipelotrichaceae* and *g__Alloprevotella* in the susceptible group; *g__Ruminococcaceae* in the control group are differentially abundant microbes (DAM) between groups (Figure 3E). Besides, the KEGG pathway analysis shows that the DAM in the susceptible group is significantly enriched for G protein−coupled receptors and Biosynthesis of 12‐, 14‐, and 16‐membered macrolides. The DAM in the control group is significantly enriched for the mRNA surveillance pathway, Renin−angiotensin system, Indole alkaloid biosynthesis, Betalain biosynthesis, and 1,1,1‐Trichloro‐2,2‐bis(4 − chlorophenyl) ethane (DDT) degradation. The DAM in the unsusceptible group is enriched for Carotenoid biosynthesis (Figure 3F).

### 3.4 Reprogramming of cerebral metabolism in ALI mice with cognitive dysfunction

**FIGURE 4:**
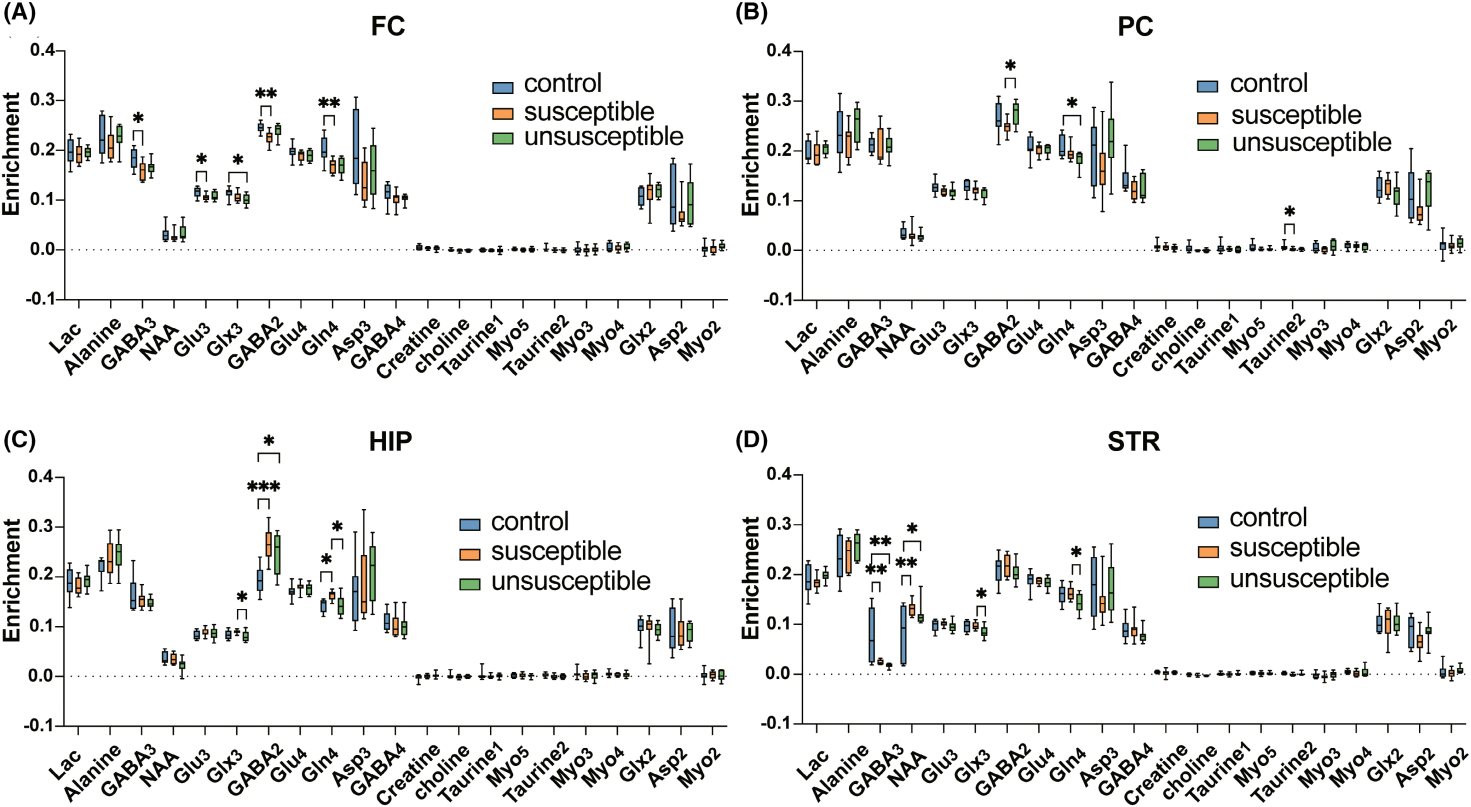
^13^C enrichments of different metabolites classified by brain regions in mice. (A–D) Box plots of metabolite ^13^C enrichments in FC, PC, HIP, and STR. Note: Asp, aspartate; GABA, γ‐aminobutyric acid; Gln, glutamine; Glu, glutamate; Glx, glutamine + glutamate; Lac, Lactic acid; Myo, Myo‐inositol; NAA, N‐acetyl aspartate; Subscript: proton signals connected with the related ^13^C positions (1 ‐ 4) in the metabolites. Note: The letters before the metabolite name represent the different brain regions, CE, cerebellum; FC, frontal cortex; HIP, hippocampus; PC, parietal cortex; PON, medulla‐pons; STR, striatum; TC, temporal cortex; TH, thalamus. The data were analyzed by one‐way ANOVA followed by Tukey's multiple comparisons test. Data are presented as the mean ± SEM. *p < 0.05; **p < 0.01; ***p < 0.001.

With the infusion of [1‐^13^C]‐glucose, we traced the metabolic process of glucose in neurons and astrocytes by assessing metabolites labeled with ^13^C at different positions.^28^ In the FC, compared with the control group, the enrichment of GABA_3_ (*p* = 0.0238), Glu_3_ (*p* = 0.035), GABA_2_ (*p* = 0.007), and Gln_4_ (*p* = 0.001) were decreased in the susceptible group, while only the Glx_3_ (overlapping signals of Gln_2_ and Gln_3_, *p* = 0.041) enrichment was decreased in the unsusceptible group (Figure 4A). In the PC, the GABA_2_ (*p* = 0.043) enrichment in the susceptible group was decreased compared with the unsusceptible group, and the Gln_4_ enrichment (*p* = 0.019) in the unsusceptible group was decreased compared with the control group (Figure 4B). In the HIP, the Glx_3_ (*p* = 0.049) enrichment differed between the two ALI groups. The GABA_2_ enrichment (*p* = 0.045) was increased in two ALI groups compared with the control group. The susceptible group had higher Gln_4_ enrichment (*p* = 0.005) than the unsusceptible and the control groups (Figure 4C). In the STR, the control group had significantly higher GABA_3_ (*p* = 0.001) and lower NAA (*p* = 0.012) than the two ALI groups. The enrichment of Gln_4_ (*p* = 0.028) and Glx_3_ (*p* = 0.034) in the susceptible group were increased compared with the unsusceptible group (Figure 4D). In addition, the Glx_3_ (*p* = 0.020) enrichment was decreased in the unsusceptible group compared with the control group in PON (Figure S2D). Taken together, these findings provide evidence that neurotransmitter metabolism, particularly GABA metabolism, is changed in various brain regions associated with cognitive function after ALI.

### 3.5 Correlation among metabolites, microbiota, and behaviors in ALI mice

**FIGURE 5:**
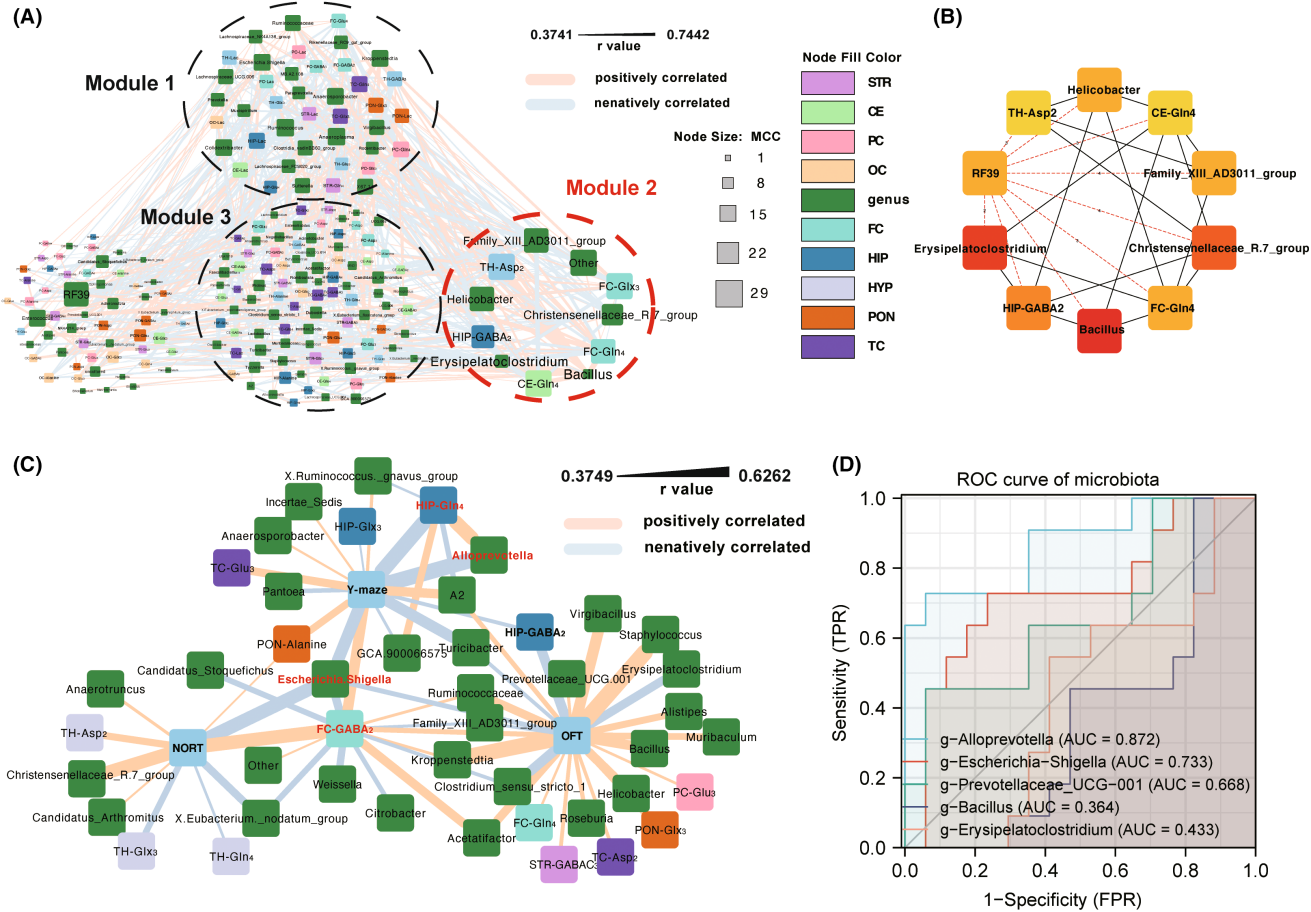
Correlation network analysis among microbiota, neurochemical enrichments, and behavioral test. (A) The Spearman test was used to assess the correlation between major neurochemical enrichments and major microbiota in the genus. Module identification of correlation network by Molecular Complex Detection (MCODE) plugins (degree ≥ 10, Node Score ≥ 0.4, K‐Core ≥ 2, max depth: 100). (B) Top 10 hub candidates identified by Maximal Clique Centrality (MCC). (C) Correlation network analysis of microbiota, neurochemical enrichments, and behavioral test. Note: Metabolites having a mean enrichment > 0.05 (Lac, Alanine, GABA_3_, Glu_3_, Glx_3_, GABA_2_, Glu_4_, Gln_4_, Asp_3_, GABA_4_, Glx_2_, and Asp_2_) and top 100 microbiotas were selected as major neurochemicals and major microbiotas to report. The spearman coefficient (*r*) was adjusted by FDR and ranged from positive (light pink edge) to negative (light blue edge). Node size is proportional to the node's MCC score, indicating its interaction value. Nodes are colored according to behavior, microbiota in the genus, and brain regions of metabolites. (D) ROC curve of major microbes.

We conducted a correlation analysis between microbiota and major neurochemicals, followed by network construction using the correlation coefficient (Figure 5A, Appendix S1). This created a highly complex network, implying that gut microbes and neurochemical metabolism may have a complicated interaction. MCODE analysis revealed three modules (Cluster Score: module 1 = 5.3, module 2 = 5, module 3 = 3.283) consisting of nodes with relatively higher local neighborhood density than the rest of the network.^29^ Interestingly, the CytoHubba analysis identified the genera in module 2 as hubs with top MCC scores^30^ (Figure 5B). *Bacillus* possesses the most interactions with neurochemicals (MCC = 29), followed by *Erysipelatoclostridium* (MCC = 27), indicating that these genera may extensively affect neurochemical metabolism in multiple brain regions. Among the neurochemicals, HIP‐GABA_2_ (MCC = 21) and FC‐Gln_4_ (MCC = 20) have the most interactions with microbiota.

We further analyzed the correlation between microbiota, neurochemicals, and behavior (Figure 5C). It is noted that a mutually interacting subnetwork consisting of *Alloprevotella*, HIP‐Gln_4_, and behavioral performance in the Y‐maze could be identified, in which behavioral performance is strongly correlated to *Alloprevotella* abundance (*r* = −0.6169) and HIP‐Gln_4_ enrichment (*r* = −0.6048), while *Alloprevotella* may also affect hippocampal glutamine metabolism (*r* = 0.5879). It is also worth noting that FC‐GABA_2_ enrichment appears as a hub of the network and is positively associated with all three behaviors (FC‐GABA_2_ to OFT, *r* = 0.4325; FC‐GABA_2_ to NORT, *r* = 0.5841; FC‐GABA_2_ to Y‐maze, *r* = 0.5313). Meanwhile, FC‐GABA_2_ enrichment also negatively correlates with the abundance of *Escherichia.Shigella* (*r* = −0.4913), which is a microbe with the highest degree in the network and negatively correlates with three behaviors (*Escherichia.Shigella* to OFT, *r* = −0.4302; *Escherichia.Shigella* to NORT, *r* = −0.5807; *Escherichia.Shigella* to Y‐maze, *r* = −0.5546). These results indicate that increased *Alloprevotella* and *Escherichia.Shigella* may modulate HIP‐astrocytes and FC‐GABAergic neurons' metabolic reprogramming in response to ALI‐induced cognitive dysfunction in mice.

We then assessed the performance of microbes in classifying the susceptible and unsusceptible groups using receiver operating characteristic (ROC) analysis (Figure 5D). We found *Alloprevotella* and *Escherichia.Shigella* with the area under the ROC curve (AUC) of 0.872 and 0.733, respectively, may serve as indicators of CD.

### 3.6 The mIPSCs and mEPSCs of CA1 and Prl were suppressed in ALI mice

**FIGURE 6:**
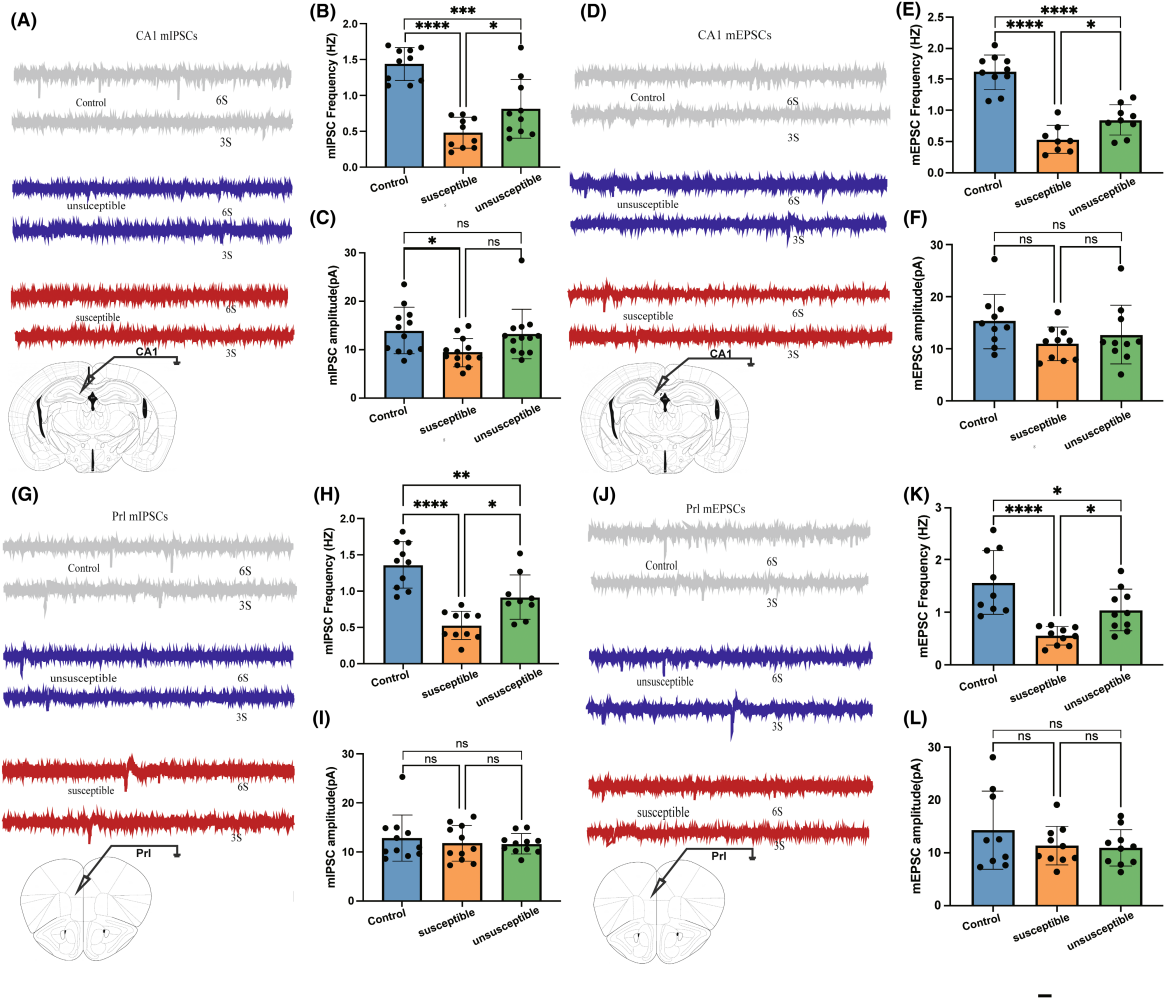
Different mIPSCs and mEPSCs in Prl and hippocampus CA1 pyramidal neurons in ALI mice. (A, D) mIPSCs (A) and mEPSCs (D) recordings of the control group, the susceptible group, and the unsusceptible group in CA1. (G, J) mIPSCs (G) and mEPSCs (J) recordings of three groups in Prl (*n* = 6). Calibration: 1 sec, 20 pA. (B, E) Frequency of mIPSCs (B) and mEPSCs (E) in CA1. (C, F) Amplitude of mIPSC (C) and mEPSC (F) in CA1(*n* = 6). (H, K) Frequency of mIPSCs (H) and mEPSC (K) in Prl. (I, L) Amplitude of mIPSCs (I) and mEPSCs (L) in Prl. *p < 0.05; **p < 0.01; ***p < 0.001; or ****p < 0.0001. Data are shown as mean ± SEM.

Network analysis highlighted neurochemical metabolism in the HIP and FC as essential nodes of the network (higher MCC scores). Meanwhile, both HIP and FC have been widely implicated in cognition. We speculated that dysregulated neurotransmitter metabolism could alter neurotransmission in these brain regions, thereby impairing the cognitive function of affected animals. In this connection, we recorded the mIPSCs and mEPSCs in CA1 and PrL. In CA1, the frequencies of mIPSCs and mEPSCs are significantly decreased in the susceptible group compared with the control group (Figure 6A–F). The frequencies were further reduced in the susceptible group compared with the unsusceptible group (Figure 6B,E). There is no difference in the amplitudes of miniature postsynaptic currents (mPSCs) between groups, except that the susceptible group has smaller amplitudes of mIPSCs compared with the control group (Figure 6C). The results showed that excitatory and inhibitory synaptic transmission can be suppressed by ALI and that cognitive impairment develops with aggravated inhibition of neurotransmission in CA1. Given that the reduction in frequencies generally indicates a presynaptic mechanism, the results are consistent with the finding that ALI causes dysregulated neurotransmitter metabolism. In addition, the decreased amplitudes of mIPSCs indicated that a postsynaptic mechanism might also be involved in ALI‐associated cognitive impairment. A similar pattern of changes in the frequencies of mIPSCs and mEPSCs was also observed in PrL (Figure 6G–K). However, the amplitudes of the mPSCs are comparable between all groups (Figure 6G,I,J,L). Taken together, these results indicate that suppressed synaptic transmission in CA1 and Prl caused by reprogrammed neurotransmitter metabolism may be the mechanism of ALI‐induced CD in mice.

## 4 DISCUSSION

Clinical studies have shown that some ALI patients develop different degrees of HE and progress to ALF, eventually leading to the need for liver transplantation or death.^31^, ^32^ TAA is widely used to develop animal models of ALI and HE,^33^, ^34^ which are believed to share similar manifestations in humans.^35^ In our study, we induced ALI in mice by multiple injections of low‐dose TAA, resulting in hepatocyte necrosis and elevated plasma ALT and AST. Clustering behavioral performance in the Y‐maze test identified spatial memory impairment in 55% (11/20) of ALI mice, similar to that observed in patients with chronic liver disease.^36^

Microbiota dysbiosis is involved in liver disease^16^, ^18^ and its complications.^37^ For example, *Ruminococcaceae* was found to be reduced in patients with HE.^38^ Transplantation with stool rich in *Ruminococcaceae* improved cognition and reduced hospitalizations in HE patients.^39^ In this study, we found that control mice have higher *Ruminococcaceae* abundance than mice with ALI. Moreover, our study concurs with the clinical trial by showing that cognitive impairment is transmissible to intact mice through transplanting stool from cognition‐impaired mice. Previous studies reported that the family and the genus level of *Erysipelotrichales*, *Erysipelatoclostridium* is positively correlated with cognitive function in Alzheimer's disease mice^40^ and clinical benefits of rifaximin in Alzheimer's disease patients.^41^ However, *Erysipelotrichaceae* correlates significantly with brain inflammation.^42^ Consistent with these reports, *Erysipelatoclostridium* and *Erysipelotrichaceae* were increased in the unsusceptible and susceptible groups, respectively. *Alloprevotella* is increased in hepatitis B and alcoholic fatty liver,^43^, ^44^ and increased *Alloprevotell*a in the duodenum could predict the risk of cirrhosis and hepatocellular carcinoma.^45^ Similarly, in our result, *Alloprevotella* is increased in the susceptible group.

Impaired brain energy metabolism and neuronal dysfunction due to glutamine accumulation‐induced swelling of astrocytes is a major mechanism of HE.^46^, ^47^ Besides, abnormal glutamine accumulation may also affect the synthesis of neurotransmitters like glutamate and GABA.^48^ Neurotransmitter alterations due to energy metabolism disorders are reported to be directly involved in the pathogenesis of HE.^49^ In vitro ^1^H‐[^13^C] NMR studies in rats have shown that the enrichments of Gln_4_ and Clu_4_ are increased in the FC of HE rats, while GABA_2_ enrichment is unchanged.^11^, ^12^ In our study, the Gln_4_ enrichment in the susceptible group is increased in subcortical regions such as HIP and STR, but not in cortical regions. This may be explained by the findings that higher ammonia uptake is observed in subcortical brain regions in MHE patients, making subcortical metabolism more sensitive to ammonia.^46^ In addition, GABA_2_ enrichment is decreased in cortical areas such as FC and PC in the susceptible group and is increased in HIP. The results are consistent with previous reports that in the rats with hyperammonemia, extracellular GABA content is increased in HIP but decreased in cortical areas.^50^

The gut microbiota has been reported to modulate neurotransmitters.^51^ However, there is no report on the relationship between gut microbiota and the metabolic dynamics of specific neurotransmitters in the brain. *Escherichia* produces GABA in the human gut.^52^ The increase in *Escherichia.Shigella* has been described as a pro‐inflammatory flora in cognitively impaired elderly^53^ and post‐operative mice with cognitive dysfunction^54^ and is negatively correlated to cognition in Alzheimer's disease patients.^55^ Consistently, we found that increased abundance of *Escherichia.Shigella* is negatively correlated with FC‐GABA_2_ and all three behaviors. *Alloprevotella* is reported to be negatively correlated to cognition in Phenylketonuria mice^56^ and related to transmitter metabolism in hippocampus.^57^ In this study, the increased *Alloprevotella* is significantly negatively correlated with Y‐maze and positively correlated to HIP‐Gln_4_. We proposed that *Alloprevotella* and *Escherichia.Shigella* could be a potential biomarker to predict cognitive impairment in ALI (AUC = 0.872 and 0.733). These findings suggest that specific microbiota correlates to metabolic dynamics of brain transmitters and behavior.

Maintenance of cognitive function depends on normal synaptic transmission. Previously reported that neurotransmission disorders are responsible for the cognitive function alteration in HE.^14^ We found that the frequencies of both mIPSCs and mEPSCs in CA1 and PrL were decreased in the susceptible group compared to the unsusceptible group. These changes were consistent with the reprogrammed metabolic kinetics, highlighting a presynaptic mechanism underlying ALI‐induced cognitive dysfunction. In addition, the correlation of behaviors, microbes, and metabolites provides a novel insight into the perspective of gut microbiota and brain metabolism for ALI‐induced cognitive dysfunction. *Escherichia.Shigella* elevation‐related decrease in FC‐GABA_2_ enrichment and *Alloprevotella* elevation‐related increase in HIP‐Gln_4_ enrichment might play roles in the cognitive dysfunction in ALI‐induced CD mice. However, the effects of specific microbes and metabolites have not been validated. The development of therapeutic strategies targeting *Escherichia.Shigella* and *Alloprevotella* have great application prospects for preventing and treating ALI‐induced CD.

## 5 CONCLUSION

This work revealed altered gut microbiota and cerebral metabolism in acute liver injury mice with and without cognitive dysfunction. *Escherichia.Shigella*‐related decreased FC‐GABA_2_ enrichment and *Alloprevotella*‐related increased HIP‐Gln_4_ enrichment might be responsible for the cognitive dysfunction in ALI mice. *Alloprevotella* and *Escherichia.Shigella* are biomarkers to differentiate ALI with and without cognitive dysfunction.

## AUTHOR CONTRIBUTIONS

TS, HD, and HX designed and drafted the manuscript. TS, HD, ZL, JX, YL, YL, WZ, FL, and JH acquired data. TS, XL, and HX analyzed the data. All authors approved the final version of the manuscript for publication.

## CONFLICT OF INTEREST

The authors declare no conflict of interest.

## Supporting information


Figure S1

Figure S2

Appendix S1

Figure Legend

## Data Availability

The data that support the findings of this study are available in the supplementary material of this article.
