# Supplementary figures and images for: Decoding the contributions of gut microbiota and cerebral metabolism in acute liver injury mice with and without cognitive dysfunction
Source: CNS Neurosci Ther. 2022 Dec 30;29(Suppl 1):31–42. doi: 10.1111/cns.14069 (PMC10314109; doi:10.1111/cns.14069)

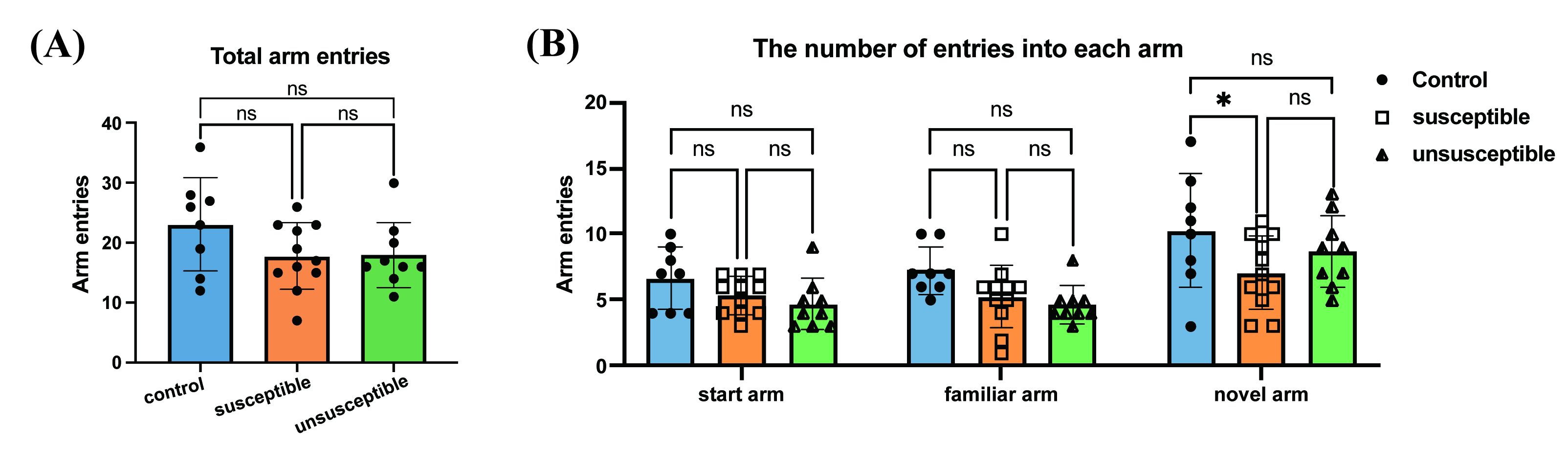

Supplement: Supplementary file 1 — Figure S1 [file CNS-29-31-s003.tif]
