# Supplementary material for: Decoding the contributions of gut microbiota and cerebral metabolism in acute liver injury mice with and without cognitive dysfunction
Source: CNS Neurosci Ther. 2022 Dec 30;29(Suppl 1):31–42. doi: 10.1111/cns.14069 (PMC10314109; doi:10.1111/cns.14069)
Supplement: Supplementary file 4 — Figure Legend [file CNS-29-31-s002.docx]

**
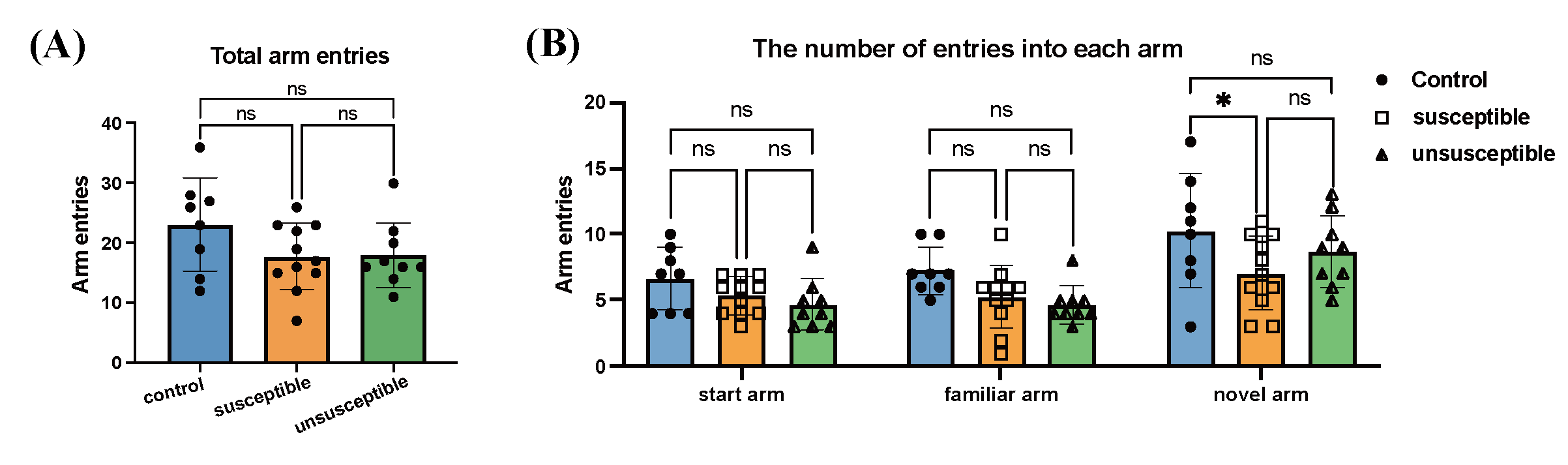
**

**Supplementary Figure 1.** **Y-maze test arm entries of ALI mice.** (A) Total arm entries (ANOVA, F_2, 25_ = 2.014, P = 0.1545). (B) The number of entries into each arm (Two-way ANOVA, Control vs. susceptible, P = 0.0196). Data are presented as the mean ± SEM. *p < 0.05.


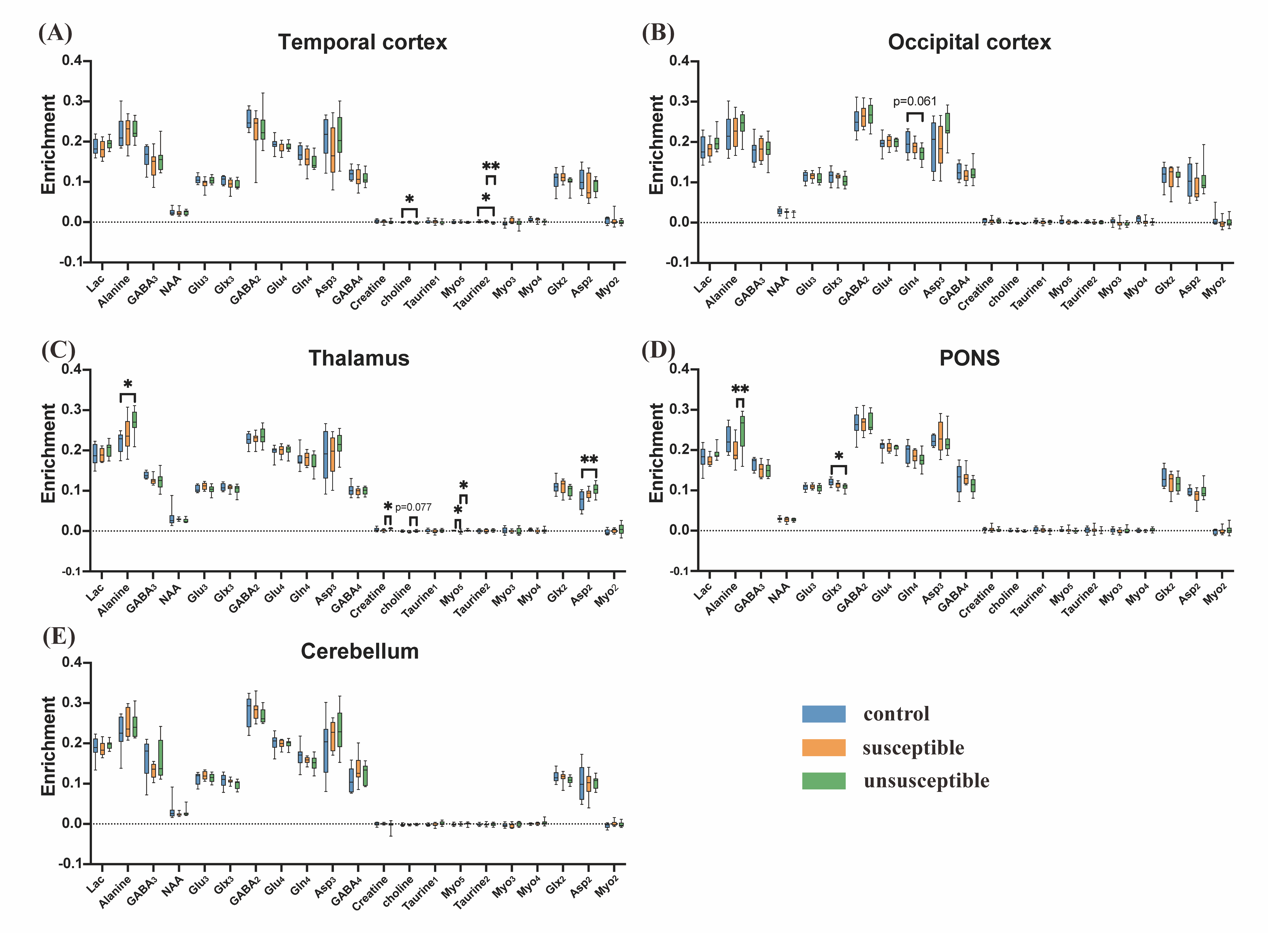


**Supplementary Figure 2. ^13^C enrichments of different metabolites classified by brain regions in mice.** (A-E) Box plots of metabolite ^13^C enrichments in TC, OC, TH, PON, and CE. Note: Lac: Lactic acid; GABA: γ-aminobutyric acid; NAA: N-acetyl aspartate; Glu: glutamate; Glx: glutamine + glutamate; Gln: glutamine; Asp: aspartate; Myo: Myo-inositol; Subscript: proton signals connected with the related ^13^C positions (1-4) in the metabolites. The data were analyzed by one-way ANOVA followed by Tukey's multiple comparisons test. Data are presented as the mean ± SEM. *p < 0.05, **p < 0.01, ***p < 0.001.
